# Indoor Air Pollution and Decision-Making Behavior: An Interdisciplinary Review

**DOI:** 10.7759/cureus.26247

**Published:** 2022-06-23

**Authors:** German Torres, Mervat Mourad, Joerg R Leheste

**Affiliations:** 1 Counseling and Clinical Psychology, Medaille College, Buffalo, USA; 2 Biomedical Sciences, New York Institute of Technology College of Osteopathic Medicine, Old Westbury, USA; 3 Clinical Specialties, New York Institute of Technology College of Osteopathic Medicine, Old Westbury, USA

**Keywords:** indoor air management, vietnam war, smoke toxicants, particulate matter, neural circuits, cognitive behavior

## Abstract

The human brain is constantly exposed to air pollutants, some of which might be disruptive or even lethal to certain neurons implicated in abstract features of cognitive function. In this review, we present new evidence from behavioral and neural studies in humans, suggesting a link between indoor fine particulate matter and decision-making behavior. To illustrate this relationship, we use qualitative sources, such as historical documents of the Vietnam War to develop hypotheses of how aerial transmission of pollutants might obstruct alternative choices during the evaluation of policy decisions. We first describe the neural circuits driving decision-making processes by addressing how neurons and their cognate receptors directly evaluate and transduce physical phenomena into sensory perceptions that allow us to decide the best course of action among competing alternatives. We then raise the possibility that indoor air pollutants might also impact cell-signaling systems outside the brain parenchyma to further obstruct the computational analysis of the social environment. We also highlight how particulate matter might be pathologically integrated into the brain to override control of sensory decisions, and thereby perturb selection of choice. These lines of research aim to extend our understanding of how inhalation of airborne particulates and toxicants in smoke, for example, might contribute to cognitive impairment and negative health outcomes.

## Introduction and background

**Figure 1:**
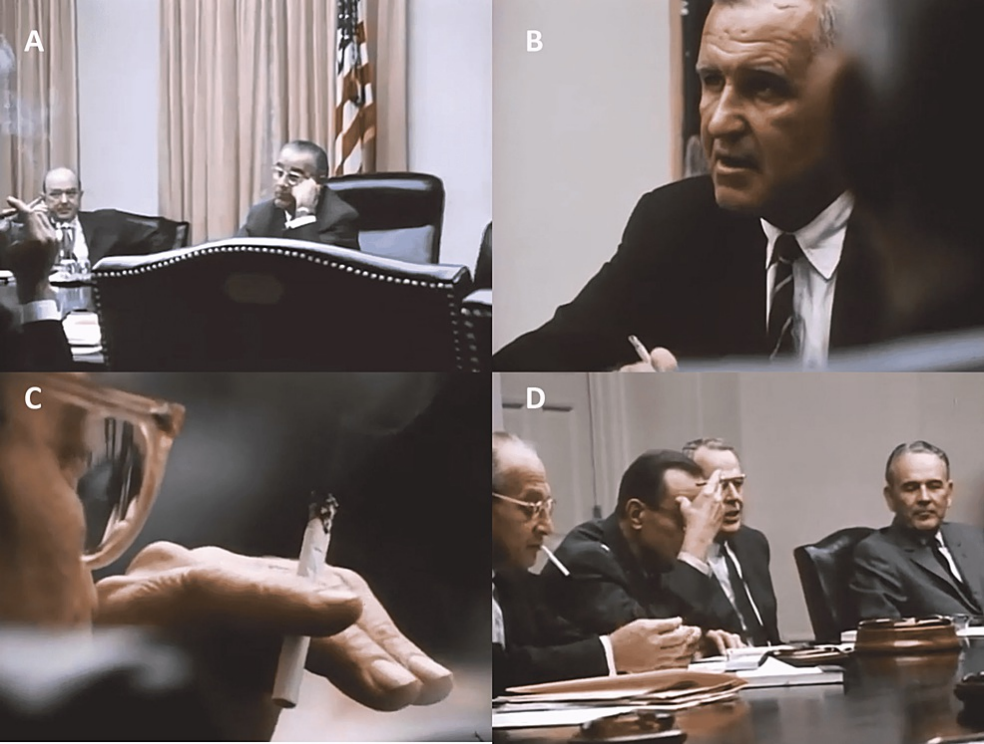
Footage from the Lyndon Baines Johnson Library. Film clips depict the President of the USA with several of his secretaries and advisors discussing lay theories of peace for ending the prolonged and violent conflict in Vietnam. The film clips also show secretaries and advisors to the President smoking cigarettes in the West Wing of the White House. We now know that increased exposure to smoke toxicants results in negative health outcomes, independent of local concentrations of other indoor air pollutants. Although pulmonary and cardiovascular obstructive diseases are traditionally associated with human exposure to smoke, smoke particulates (> 1 µm) also injure large-scale brain networks that drive decision-making behavior. Thus, high levels of smoke plumes, for example, could have increased the risk of cognitive impairment by directly reducing LBJ’s cortical blood flow. Blood flow as measured by phase-contrast MRIs is an index of local neural activity. (A) LBJ and Secretary of State Dean Rusk; (B) Creighton W. Abrams United States Army General; (C) Walt Rostow National Security Advisor to LBJ; (D) from left to right: Walt Rostow, Earle Wheeler (Chairman of the Joint Chiefs of Staff), Clark M. Clifford (Secretary of Defense), and Maxwell D. Taylor (Chairman of the Foreign Intelligence Advisory Board). Images used for this composite are available through the photo archive of the LBJ Library (http://www.lbjlibrary.net/collections/photo-archive.html) through the public domain and are free for use by anyone for any purpose without restriction under copyright law.

In October 1968, President Lyndon Baines Johnson (LBJ) attended several cabinet meetings with a conclave of top advisers to discuss strategies related to the ongoing war in Southeast Asia. Vietnam now occupies most of the president’s time and energy and the quagmire of the conflict is rapidly hogging both his political standing and national treasure to the point that he can no longer pursue the Great Society Programs he once bestowed upon his presidency. Footage from the LBJ Library (The President: MP901) depicts several cabinet meetings in session. Additionally, narratives from these advisory meetings reveal fundamental discussions about conflict resolution and lay theories of peace. The MP901 footage also shows the meetings being held indoors in airtight, insulated buildings with cabinet secretaries and advisors smoking cigarettes and cigars while forging strategies for ending the war (Figures 1A-1D).

Although not readily apparent in 1968, epidemiologic evidence, consistent with experimental evidence gathered ever since shows that tobacco particulates and aerial emissions from heating and cooling systems might impair brain function. As the brain provides the anatomical skeleton for cognition and behavior, it is conceivable that exposure to particulate matter less than 2.5 µm (PM2.5) in aerodynamic diameter might have affected diverse aspects of LBJ’s brain states, including, sensory processing, sympathetic arousal, cognitive function, and/or action selection. In doing so, policy-relevant decisions about Vietnam could have been negatively influenced by ambient conditions previously thought to be innocuous. Against this background, this review will examine how decision-making processes and similar cognitive functions might be affected by indoor air pollution. This is of singular importance as we now spend most of our working and leisure time indoors, typically isolated from fluctuations in temperature and humidity, airflow patterns, solar irradiance, and other daily and seasonal climatic forces that have shaped the evolution of hominins. This scientific hypothesis has drawn attention from researchers in both the social and natural sciences who share common interests in understanding how sensory-signaling neurons and environmental cues coalesce to form the basis of human experience. Thus, political scientists are interested in evaluating past and current human behavior so that they can better forecast how individuals or groups make decisions and exercise power. Neuroscientists, on the other hand, are interested in understanding how synapses connect neurons in the brain to build the circuits that enable cognitive behavior. Of note, this review is not comprehensive; we only use selected articles from the recent literature to illustrate relevant points of interest.

## Review

**Figure 2:**
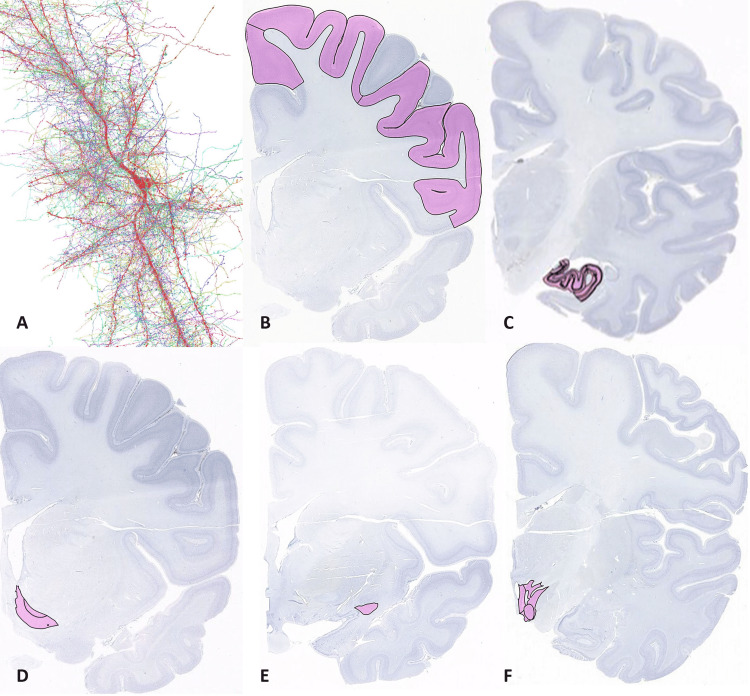
Neural Circuits and Cognition. The cognitive faculties of the human brain are underpinned by intricate and highly interconnected neural circuits. (A): Neurons from the following brain areas (in pink color) are thought to broadcast local chemical signals to a wider brain network to generate a subjective computational analysis of the social environment; (B) Cortex: different regions of the cortex (e.g., dorsal prefrontal cortex and orbitofrontal cortex) have specific cognitive functions and distinct computational properties; (C) hippocampus; (D) nucleus accumbens; (E) amygdala; (F) hypothalamus. These chemical signals are likely correlated with diverse aspects of behavior, including decision-making, declarative memory, intrinsic reward, anxiety, fear learning, and homophily cooperation, respectively. Obviously, chemical signals from other brain areas (e.g., periaqueductal gray and anterior insula) as well as peripheral nerves interact with the above neural circuits to provide complementary inputs to discern the best course of action among competing alternatives [1]. Human nerve cells (A) are from Lichtman Lab/Harvard University, Connectomics Team//Google. (B-F) Coronal brain sections (Nissl stained, ~14667-14712 µm) are from a 34-year-old male. Image Credit: Allen Institute (Interactive Atlas Viewer). Content for research and academic publication may be used without further permission as stated (https://alleninstitute.org/legal/terms-use/).

There is a consensus that behavior is generated by the chemical activity of synapses that connect neurons. These anatomical infrastructures are populated by neurotransmitters and RNA species that interact with each other and the environment to yield a subjective sensory experience. Building on this tenet, it is thought that cognitive processes such as perception, attention, memory, and emotion are derived from complex patterns of chemical activity represented by features of brain organization and structure (Figures 2A-2F).

For example, the neural circuits involved in anxiety (e.g., panic disorder), an emotional experience linked to stress or adversity, appear to be anatomically co-localized to the frontal cortex, the extended amygdala, and the periaqueductal gray area (PGAG of the brain [2]. The PAG is a midbrain structure whose neurons process negative emotions such as perceived personal threats to enhance the flexibility and evolutionary fitness of defensive behaviors [3]. Continuing with this parcellation of brain function, neural circuits mediating episodic and working memory reside within the hippocampus and the angular gyrus; brain regions that when spiked with a sensory cue generate a personal memory trace of the past and a predictive valence of the future [4]. These two examples collectively suggest that neurons interact with each other and nonneuronal cell types (e.g., astrocytes) at specific loci of the brain to generate complex behaviors with social threat learning undertones [5]. The fact that diverse aspects of brain states are grounded in connectivity and activity patterns of neurons also suggests that disturbances in the structural and functional integrity of synapses, for example, can lead to flaws in the computational mechanisms associated with risk-benefit analysis. Indeed, if synapses become dysfunctional or are lost after brain injury, the ability to retrieve information from memory and the use of this information to make decisions is often impaired [6,7].

The microanatomy for decision-making processes in the brain seems even more complex as the genes and molecules coding for these particular behaviors may not be found in a single place but are likely distributed across the cerebral cortex through intricate cell networks. The cerebral cortex is a highly convoluted brain structure, organized along with six columnar and laminar layers that vary markedly in terms of gene expression, cell type, and functional connectivity (i.e., input and output connections). The contemporary theory posits that this brain region is unique in that it not only responds broadly to sensory cues but also segregates and selectively directs different types of sensory information to the striatum (i.e., cortico-striatal connection). For example, neurons of the striatum respond to chemical signals from the orbitofrontal cortex in a region-specific manner: the ventral striatum (or nucleus accumbens) is associated with reward or hedonistic behaviors; the caudate nucleus with cognition; and the putamen with voluntary motor control [8,9]. These behaviors subsequently add complexity to decision-making processes as they modulate how the brain decides the best course of action among competing alternatives. Thus, computational properties of making a decision appear to emerge from the canonical circuits of the six-layered cerebral cortex along with highly associative areas in the striatum, extended amygdala, and hippocampus [10-13]. However, there is now considerable evidence that additional neural systems are recruited when decisions are made in the context of social interactions. Some of these effector areas include the thalamus, a sensor relay station, whose neurons broadcast chemical signals to cortical and subcortical synapses to coordinate perceptual decisions [14]. The hypothalamus also contributes to shaping cognitive behavior (e.g., social memory) through the actions of oxytocin, a hormone produced in magnocellular neurons of the anterior hypothalamus [15,16]. This neuropeptide hormone promotes social behavior, including homophily cooperation, and it is therefore an additional signaling pathway in the computational architecture that encodes trust, reciprocity, altruism, and fairness; human intrinsic values that affect decision-making processes [17]. Thus, axonal inputs and outputs from the hypothalamus are directed to-and-from the hippocampus and thalamus to build a functional circuit that interprets and translates sensory information into a socially learned behavior.

Box 1: Open questions

What additional factors might affect the neural mechanisms underlying decision-related processes? As discussed earlier, a myriad of neurons operates in tandem to generate cognitive behavior. However, it is becoming clear that behavior, in general, can be altered by developmental processes imprinted during the formation of the central nervous system (CNS). This imprinting or epigenetic phenomenon affects gene activity and expression without altering the underlying DNA sequence; a molecular phenomenon that is both mitotically stable and meiotically inherited over generations [18]. Thus, epigenetics provides the molecular mechanisms for life experiences to directly affect basic cell processes linked to behavior [19]. Although epigenetic phenomena are complex and influenced by DNA variants, cell type, gender, and age, it is conceivable that action phases (i.e., sensory input and perception, item evaluation, and action selection) that define decision-making behavior can become programmed in one individual and later inherited in the synaptic diagram of another. It may be the case that some of the decisions made by LBJ’s nervous system during the Vietnam War could have been influenced independently by the president’s intentions or awareness. The possibility that implicit (unconscious) decision bias of the past might affect the choice assessment of the future is an important factor to consider when studying the signatures of internal brain states associated with the computational analysis of the social environment [20]. Reconstructing this relationship, in turn, is critical to understanding the evolution of our relationship with the physical environment.

Box 2: Open questions

What additional factors might affect the neural mechanisms underlying decision-related processes? Neurons use transmitters to process information and employ this inference to make decisions. Indeed, serotonin (5-hydroxytryptamine or 5-HT) and dopamine (DA) molecules in the striatum (caudate nucleus and putamen) participate in perceptual decision-making processes, thus shaping how humans perceive and act upon their sensory experiences [21]. The fact that neurotransmitter release drives sensory experiences raises the question as to whether additional sources of 5-HT and DA may affect the computational analysis of perceptual decisions. A potential source for these molecules is the trillions of microbes that reside within the human gastrointestinal tract. Indeed, microbes can produce 5-HT, DA, glutamate, γ-aminobutyric acid (GABA) and other chemical ligands used by neurons to run a functioning brain [22-24]. As these ligands and their metabolic by-products can be absorbed into the bloodstream and delivered to the brain across circumventricular areas (e.g., organum vasculosum of the lamina terminalis), cranial nerves (e.g., vagus nerve X), and/or meninges (e.g., dural venous sinuses), it is conceivable that additional 5-HT or DA levels may disrupt the balance of excitatory and inhibitory mechanisms that determine the strength of synaptic transmission [25,26]. In this context, disruptions in the delicate balance of microbes within the human gut are associated with neurological disorders, and epidemiological data indicate that viral and bacterial tropism leads to psychiatric syndromes, including psychosis, delirium, and major depressive disorders [27,28]. Overall, these findings imply that in addition to epigenetics, microbes might also contribute to shaping decision-making behavior by determining which synapse gets reinforced and which one gets weaker via the amount of neurotransmitter they produce during cell-specific action. In summary, microbes provide an additional source of chemical ligands that can be deployed in analogous roles to those molecules made in the CNS to affect the sensory experience and ultimately behavioral output.

Indoor air pollution

**Figure 3:**
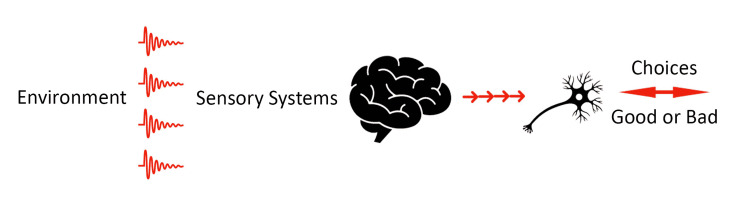
Schematic Diagram Depicts Progressive Action Phases Thought to be Involved in the Generation of Decision-Making Behavior. Continuous signals from the environment are perceived by sensory systems which are then processed, coded, and experienced as segmented units by the brain. These experiences evoke complex patterns of neural activity which are then transmitted to cortical and subcortical cell type-specific areas. Some experiences such as personal, physical threats are most likely processed within neural circuits of the eyes (e.g., VG3 amacrine cell-signaling), not the brain [29]. Thus, some decisions are made in single automatic trials which are crucial for human and animal survival. Decision-making behavior is influenced by internal states (e.g., health status and reward values), personal evolutionary history (e.g., genetics and epigenetics), the behavior of others around us, and likely by the genes that those individuals carry. As a result, people’s choices do not always perfectly reflect the true state of the local environment. The figure was drawn and edited by Dr. German Torres and Dr. Joerg R. Leheste.

As discussed earlier, personal or grouped decisions generally consist of distinct action phases that are sequentially implemented depending on the behavioral context and goals (Figure 3).

Thus, socio-political events such as the war in Vietnam were likely associated with heightened anxiety states which might have impacted specific decision variables related to conflict resolution. Although anxiety-related phenomena certainly interfere with everyday decisions, there is now evidence that seemingly innocuous factors such as indoor air pollutants might also have a significant cumulative effect on cell types driving the computation of choice [30]. Most notably are fine PM2.5 which appear to be well-established risk factors for cardiovascular and pulmonary obstructive diseases [31,32].

**Figure 4:**
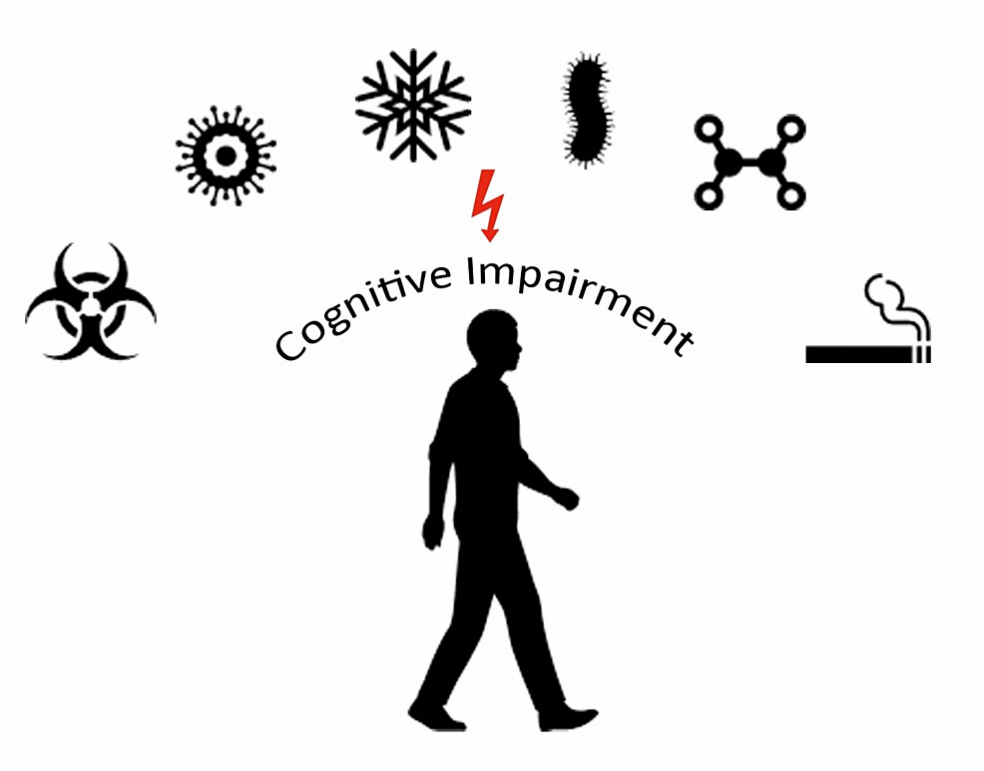
Schematic Diagram Depicts How High Levels of Particulate Matter Might Contribute to Cognitive Impairment and Motor Disability in Humans. PM2.5 enters the brain via respiratory and olfactory epithelia, the integumentary system, and visual and/or auditory afferent pathways. Exposure to sulfates, nitrates, and/or volatile organic compounds negatively affects the electrical and chemical signals of the functioning neuron. This exposure is exacerbated by prolonged indoor living, inadequate air filtration, droplet shedding, and exhalation of gut bioeffluents, including those produced during talking and coughing. As a result, indoor pollution levels progressively distort people’s behavior creating situations where, in extreme cases, everyone selects exactly the same choice (Good or Bad) at the expense of rational reasoning and evidence from multiple sources. The figure was drawn and edited by Dr. German Torres and Dr. Joerg R. Leheste.

We now spend most of our time inside homes, public buildings, and/or vehicles. This stay-indoor trend, in particular, has become more prevalent as the current COVID-19 pandemic has expanded exponentially across the USA [33]. Although the commonality of this trend is now beginning to be recognized, the biological consequences of such novel features of human behavior remain incompletely understood. Nor do we fully understand why high levels of indoor air pollution, most notably fine PM2.5, are at least somewhat specific to neurons [34,35]. This raises the question of whether chronic levels of PM2.5 contribute to cognitive impairment and motor disability and, if so, what measures might be taken to minimize these risk factors [36-38]. Against this background, we now suspect that poor ventilation indoor, enclosed spaces can lead to the accumulation of bioaerosols (e.g., fungal and bacterial droplets), smoke-related particles (e.g., nitrosamines, cadmium), environmental gases (e.g., ozone, carbon monoxide, nitrogen dioxide), and other particulate matter (e.g., molds and mycotoxins) that when ingested over time causes substantial organismal stress (Figure 4) [39-44].

More specifically, epidemiological studies suggest that exposure to PM2.5 can build sufficiently to affect neural circuits driving particular behaviors or pathologies [45-48]. Although these correlational studies do not reveal causal mechanisms, they are consistent with the tenet that exposure to smoke-related toxins, for instance, can lead to allergic and/or inflammatory diseases of the upper and lower respiratory tract [49,50]. Thus, inhalation of airborne particulates can in principle affect interconnected synapses in the cerebral cortex by interfering with the processing of information about the external environment or the transduction of physical phenomena (e.g., neurotransmitter concentrations) into sensory perception. Regardless, these atmospheric studies are beginning to provide a basis for understanding how exposure to PM2.5 can affect innate, evolutionary conserved computational properties related to trust, reciprocity, altruism, fairness, revenge, and/or competition.

Humans base decision-making processes on available sensory evidence, evaluation of options, and choice selection from a set of alternatives [51]. As mentioned above, these specific computation steps require the recruitment of several neural systems with different anatomical, physiological, connective, and behavioral backgrounds [52-56]. However, the brain is not an isolated organ, it is also part of the bilateral body plan. Thus, sensory signals from the periphery (e.g., the gastrointestinal tract) provide additional and complementary evidence to a wider brain network, including cortical and subcortical neurons that specifically compute and yield a categorical judgment. This hypothesis is supported by findings, for example, that nerve cells in the PAG link negative emotions with the autonomic (e.g., sympathetic nervous system; the fight-or-flight response), endocrine (e.g., adrenal hormones; glucocorticoids), and immune (e.g., cytokines; interleukin 1β) systems to facilitate responses to personal threats [57,58]. Of interest, increases in testosterone levels, a steroid sex hormone produced in Leydig cells (of the testes) appear to reduce social generosity in economic decision paradigms through cortical (e.g., the insula) and subcortical (e.g., the striatum) mechanisms [59]. Thus, the brain along with the rest of the bilateral body plan are brought together to perform specific computation analysis to discern the best course of action.

The fact that humans combine chemical signals from diverse sources during evaluation and choice dilemmas reveals a complex landscape not conducive to generalization. These chemical signals are, in turn, influenced by our personal history (e.g., epigenetic biases), current health status, and future context goals. Thus, although there is evidence for a connection between exposure to PM2.5 and impaired cognitive function, uncovering the underlying mechanisms of this connection can be difficult (see below).

Given that we spend up to 90% of our time indoors, many of our most important decisions are made in airtight, enclosed spaces with poor air quality, as in the case of LBJ where tobacco particulates passively permeated cabinet meetings. Tobacco smoke is the source of several air pollutants and owing to their systemic activity and long persistence, PM2.5 from tobacco smoking deteriorates indoor air quality often leading to cell and tissue hypoxia, and in extreme cases, chronic lung disease [50]. As the human brain is a highly oxidative organ, oxygen levels are physiologically maintained within a critical threshold range in accordance with region-specific brain activity. Thus, if the oxygen supply is interrupted or diminished by environmental pollutants, certain neurons may become susceptible to death receptor-mediated apoptosis or necrosis. Indeed, given the critical role of the orbitofrontal cortex and hippocampal subregions (e.g., CA2 fields) in various learning and memory domains, it is thought that limited oxygen availability to these brain regions may result in impaired cognitive function [60]. This is not surprising as our brains are not wired to do complex thinking such as planning, self-awareness, or making choices in times of organismal stress. It is conceivable, therefore, that indoor pollutants may also damage neurons or cull their fine (axonal) connections to modify synaptic efficacy and statistically degrade cognitive function and action. Indeed, insights into air indoor chemistry reveal that exposure to PM2.5 can compromise brain health and well-being [45,61-63]. Several broad patterns have emerged from these studies.

First, although thousands of pollutants are inhaled daily, it is not yet clear which particulate, in general, has the potential to harm the brain. It is almost certain, however, that if there is long-term exposure to high levels of PM2.5, various air pollutants might be harmful to neurons, at least indirectly. For instance, cognitive performance in general declines when CO_2_ levels build up to >5%; 5000 ppm [60,64]. Other PM2.5 thought to be harmful to the brain include, paraformaldehyde, nitrogen dioxide, and hydroxyl (OH) radicals; all of which have the potential to affect a continuum of neural functions from basic processes involved in perception and action to more abstract features of human cognition such as decision-making processes [65]. Second, airborne pollutants, gaseous organic compounds such as sulfur dioxide, nitrogen oxide, and ammonia (NH_3_) which are precursors to PM2.5, emanate not only from indoor sites but also from humans.

That is, human bodies are sources of bioeffluents as well. People breathe out CO_2_ and NH_3_ all the time and metabolites from the gut microbiome can be transmitted horizontally, a process that allows individual by-products to mix with other PM2.5 to acquire new functions in the bilateral body plan [66]. The fact that people exchange PM2.5 in rooms with poor ventilation, typical of enclosed spaces, suggests that cognitive behavior can be altered by circulating respiratory droplets and aerosols. And third, it is not well-understood how airborne pollutants are pathologically integrated into the brain to override control of sensory decisions, and thereby perturb selection of choice. There are several hypotheses about how PM2.5 might be harmful to computational neurons. As mentioned earlier, increased exposure to CO_2_ can in itself result in hypoxia, independent of other pollutants [64]. Hypoxia is known to cause brain injury and this would explain how certain symptoms such as delirium, agitation, and confusion are manifested when CO_2_ levels are high [67]. Other neurological mechanisms of air pollutants include inflammation of the brain parenchyma.

Neuroinflammation is known to activate microglial cells, the resident macrophages of the CNS, and this activation degrades memory traces in the hippocampus, a pathological event associated with weakening or loss of synapses in the mouse brain [68]. Air pollutants-related immunological deficits and inflammatory responses might also disrupt normal circadian rhythms. We live predominantly indoors with bright fluorescent lighting and food nutrients available 24/7 h, which might interfere with the circadian oscillations of genes and proteins in the brain and bilateral body plan [69]. Indeed, epidemiological data suggest that circadian disruption increases the risk of metabolic, psychiatric, and oncological pathologies, particularly in people who live close to one another [70]. Thus, proximity (~<0.5 m distance) to airborne pollutants or proximity (~1-2 m distance) to disease-laden social networks influence not only health outcomes but also how people in closed spaces tend to think and behave [71,72].

Obviously, air pollutants might also degrade decision-making behavior through indirect and subtle underlying mechanisms including reduced sunlight exposure (due to indoor living) which would affect the synthesis of vitamin D levels (cholecalciferol, vitamin D3; and ergocalciferol, vitamin D2) from the skin. In this context, vitamin D appears to play a significant role in mammalian brain development, in the transmission of chemical signals across synapses and modulation of the adaptive and innate immune systems [52]. From these data, we can surmise then that cumulative exposure to PM2.5 can be physiologically disruptive or even lethal to certain vulnerable neurons. However, we cannot ignore the possibility that the site of action for pollutants may not entirely be in the brain but rather in the bilateral body plan as well. For instance, although the trillions of microbes in the human gut are thought to have a profound effect on the brain and might be tied to a whole host of peripheral pathologies we do not know what the health repercussions of airborne pollutants are for bacteria that reside on us and live within us [25]. Also, we do not know whether exposure to PM2.5 affects the risk for disease development in subsequent filial generations. In this context, women exposed to environmental toxins have been reported to birth children with poor health outcomes including obesity, insulin resistance, and behavioral deficits [73]. The intersection of airborne pollutants, viruses, gut microbes, and epigenetics is yet to be addressed in public health (e.g., building codes), despite growing evidence that long-term exposure to indoor air pollution results in negative health outcomes [74-76].

## Conclusions

The human brain and bilateral body plan are constantly exposed to cumulative PM2.5 through multiple routes. Although the pathophysiological consequences of indoor pollutants are poorly understood, it is becoming clear that high levels of particulates might contribute to decision-making impairment. We have illustrated the relationship between the aerial transmission of PM2.5 and neurons implicated in tactical decisions by describing the geopolitical decisions that came from LBJ’s rationale for intensifying the air and ground war strategies in Vietnam. As noted, the president and his cabinet secretaries spent considerable time in enclosed spaces with poor air filtration and inhaling tobacco PM2.5 while discussing conditions in Vietnam that were growing increasingly untenable. It is almost certain that smoke particulates not only exacerbated respiratory, allergic, and/or inflammatory conditions of LBJ but also degraded cognitive functions associated with decision-making behavior. Indeed, we now recognize that PM2.5 directly and indirectly impinges upon striatal (monoamine and catecholamine) cell-signaling and cortical blood flow to impact computational mechanisms that affect human perception and decision activity. Obviously, we do not know for certain if the fog of war in Vietnam was attributed to plumes of PM2.5, but it is tempting to speculate that some marginal damage to brain function could have been attributed to aberrant particulates and toxicants in smoke. The impact of PM2.5 on neural-signaling activity is beginning to be addressed despite the complexity of particulate matter as it moves through buildings and homes. For instance, PM2.5 varies by orders of magnitude, exposure to pollutants varies according to sources, and individual proximity to sources and persistence of indoor particles also varies greatly. Accordingly, abatement of this health issue will require more than a singular focus on disinfecting surfaces and must include effective treatments, including proper air filtration and ventilation, germicidal ultraviolet light procedures, and air quality monitoring. In general, these conclusions shed light on the pervasiveness, candidate causes, and health consequences of indoor air pollution.
